# Characteristics of the elderly who do not visit primary care physicians

**DOI:** 10.1186/2045-4015-2-7

**Published:** 2013-02-20

**Authors:** Nira Eshel, Raanan Raz, Gabriel Chodick, Michal Guindy

**Affiliations:** 1 Maccabi Healthcare Services, Department of Family Medicine, Hadassah Haktana clinic, 6 Chile St, Jerusalem, 9683204, Israel; 2 Maccabi Healthcare Services, Medical Informatics Department, Tel-Aviv, Israel; 3 Maccabi Healthcare Services, Central District, Tel-Aviv, Israel

**Keywords:** Primary care, Chronic disease, Community medicine, Geriatrics, Public health

## Abstract

**Background:**

Health care quality indicators encourage outreach programs for screening the elderly who do not voluntarily visit their primary care physician (PCP). The characteristics of this population, however, have never been rigorously studied. The aim of this study was therefore to characterize the demography and health status of the elderly who do not visit PCPs.

**Methods:**

A retrospective database study was carried out in the central district of Maccabi Healthcare Services (MHS) in Israel. People aged 65–100 years were included, excluding those who were registered for home-care treatment. The elderly who did not initiate a PCP visit during October 2007–October 2009 were compared to those who visited a PCP during this period, in terms of demographic characteristics, health services utilization, and major chronic diseases, using the computerized MHS database.

**Results:**

The study population consisted of 32,523 elderly, 1663 (5.1%) of whom had not visited PCP for at least two years (October 2007 – October 2009). The non-consulters were, on average, older, included more women and tended to have lower socio-economic class. They had fewer hospitalizations, used less prescribed medications, consulted secondary physicians less frequently and had less laboratory tests and imaging. In line with these findings, only 29% of the non-consulters were diagnosed with a chronic condition, compared with 91% of consulters.

**Conclusion:**

Our findings suggest that as a group, the older Israelis who do not initiate PCP visits are healthier than those who do. Given the high workload of PCPs in Israel, these findings do not support investing considerable efforts in reaching out to the elderly who do not voluntarily initiate PCP visits.

## Background

Current trends in community medicine promote preventive and proactive medicine, and use quality indicators to evaluate and quantify population screening and treatment [1]. These measures encourage primary care physicians (PCPs, comprising family physicians, general practitioners, and internists) to reach out to older adults who do not voluntarily initiate visits.

The literature on community health services is rife with studies on high-cost frequent consulters [2,3], but the health status of people who do not visit PCPs is understudied, probably due to lack of interest and lack of data. The few papers that did study this issue date back to the 1980s and 1990s, and do not offer clear conclusions [4-8]. The objective of the present study was to determine the demographic and clinical characteristics of the elderly who do not visit a PCP for extended time periods (at least two years), using a large population-based computerized database.

## Methods

The study was conducted in Maccabi Healthcare Services (MHS), the second largest health maintenance organization in Israel, which insures nearly two million citizens (25% of the population) countrywide. The study population included all MHS members aged 65–100 years in the central district, except for those who were registered with the MHS home-care unit, who receive treatment at home and do not require PCP visits. The elderly residing in retirement homes or hospitalized in geriatric institutions were included in the analysis, as MHS continues to cover their health expenses [9].

We classified each person as a consulter or a non-consulter, according to whether he/she visited a PCP during the two years ending in October 2009. Demographic data, along with data on utilization of additional health services and major chronic diseases, were extracted from the computerized MHS database for a period of one year, ending in October 2009.

Diagnoses of chronic diseases were extracted from MHS computerized disease registries, described in detail elsewhere [10,11]. Socio-economic status (SES) was estimated from data taken from the Central Bureau of Statistics on the general neighborhood SES [12]. This measure is based on the 1995 population and housing census, and takes into account financial resources, housing, home appliances, motorization level, education, and employment. This SES measure was grouped into three categories: low, medium, and high. Univariate tests and logistic regression analysis were used to detect associations with clinical and demographic characteristics. The study was approved by the IRB of Assuta Medical Center, Tel Aviv (reference no. 2009065).

## Results

The study population included 32,523 elderly people, with mean age of 74.9 (SD: 7.2), 55.4% women, and 72.0% married. At least one chronic disease was documented for 88% of this population, with 30% having a documented heart disease, 25% diabetes, 69% hypertension, and 15% had cancer in the present or in the past.

Among the people included in the study, 1663 (5.1%) were non-consulters by our definition, i.e., they did not visit a PCP during the two-year period we inspected. The non-consulters comprised a higher percentage of women (61.3% vs. 55.0% in the consulters group) and non-married people, and were older on average (78.1±9.0 vs. 74.7±7.0, *p* < 0.001). A full comparison of the demographic characteristics of the groups is presented in Table 1.

**Table 1:** Demographic characteristics of consulters vs. non-consulters

		**N**	**% (n) from Non-consulters**	**% (n) from consulters**	***p***
**Gender**	Male	14,519	39 (644)	45 (13,875)	<0.001
	Female	18,004	61 (1019)	55 (16,985)	
**Age group**	65–70	11,603	28 (472)	36 (11,131)	<0.001
	71–75	8,301	18 (297)	26 (8,004)	
	76–80	5,664	16 (257)	17 (5,407)	
	81–85	4,027	15 (247)	12 (3,780)	
	86–90	2,237	15 (250)	7 (1,987)	
	91–100	691	8 (140)	2 (551)	
**Marital Status**	Married	23,420	49 (821)	73 (22,599)	<0.001
	Bachelor	3,340	18 (294)	10 (3,046)	
	Widowed	2,658	16 (263)	8 (2,395)	
	Divorced	2,124	10 (161)	6 (1,963)	
	Unknown	981	7 (124)	3 (857)	
**Socioeconomic status**	Low	6,232	25 (408)	19 (5,824)	<0.001
	Medium	9,169	33 (553)	28 (8,616)	
	High	15,714	38 (628)	49 (15,086)	
	Unknown	1,408	4 (74)	4 (1,334)	

When examining health services other than PCP visits, including hospitalizations (defined as staying at least one night in hospital), using other hospital services (such as emergency room visits, outpatient clinic visit, special tests, or dialysis), imaging services, lab tests, purchase of prescribed medications, and secondary doctor visits, the non-consulters also demonstrated significantly reduced utilization. In addition, non-consulters had substantially lower rates of documented chronic diseases (29%) in comparison to consulters (91%; Table 2).

**Table 2:** Morbidity indicators and health services utilization by consulters vs. non-consulters, during a period of one year

	**% from**	**% from Consulters**	***p***
	**Non-consulters**		
**Hospitalization**	9	14	<0.001
**Other hospital services¹**	18	70	<0.001
**Imaging**	54	97	<0.001
**Laboratory tests**	25	91	<0.001
**Prescribed medication**	15	97	<0.001
**Secondary doctor visits²**	30	97	<0.001
**Any service utilized**	66	99.8	<0.001
**Chronic disease**^**3**^	29	91	<0.001
**Heart disease**	14	31	<0.001
**Diabetes**	13	26	<0.001
**Hypertension**	36	71	<0.001
**History of cancer**	7	15	<0.001

^1^ Including outpatient clinics and emergency room visits.

^2^ Any doctor that is not a family doctor, general practitioner, or internist.

^3^ Defined by the Israel Ministry of Health [13] as at least one of the following: (a) Diagnoses of at least one disease from a fixed list, (b) Continued treatment with at least one drug from a fixed list, or (c) use at least one prescribed medication for a period of 6 months or more.

In order to find independent predictors for non-consulting, a multivariable model that included age, socioeconomic status, gender, marital status, and presence of a chronic disease was calculated. Older age, lower socioeconomic status, female gender, and not being married were all found to be independently associated with higher probability for non-consulting. On the other hand, having a chronic disease was independently associated with a 37-fold decreased chance for non-consulting (odds ratio = 0.027; Table 3).

**Table 3:** Associations with non-consulting in a multivariable model

		**N**	Odds Ratio* (95% CI)	***p***
**Age Group**	**65**–**70**	11,603	Ref.	
	**71**–**75**	8,301	1.3 (1.1–1.6)	0.001
	**76**–**80**	5,664	2.3 (1.91–2.8)	<0.001
	**81**–**85**	4,027	3.6 (3.0–4.4)	<0.001
	**86**–**90**	2,237	7.8 (6.3–9.6)	<0.001
	**91**–**100**	691	16.4 (12.5–21.6)	<0.001
**SES**	**Low**	6,232	Ref.	
	**Medium**	9,169	0.9 (0.8–1.04)	0.14
	**High**	15,714	0.6 (0.5–0.7)	<0.001
	**Unknown**	1,408	0.7 (0.5–0.99)	0.20
**Gender**	**Male**	14,519	Ref.	
	**Female**	18,004	1.2 (1.1–1.4)	0.001
**Chronic disease**	**No**	3,878	Ref.	
	**Yes**	28,645	0.027 (0.024–0.031)	<0.001
**Marital status**	**Married**	23,420	Ref.	
	**Single**	3,340	1.9 (1.6–2.3)	<0.001
	**Widowed**	2,658	1.7 (1.4–2.1)	<0.001
	**Divorced**	2,124	1.8 (1.5–2.2)	<0.001
	**Unknown**	981	2.2 (1.7–2.8)	<0.001

CI – Confidence interval, SES – Socioeconomic status.

* Odds ratio for non-consulting, mutually adjusted for all parameters in the model.

## Discussion

The present study examined the characteristics of the elderly who did not consult a PCP for at least two years, in the central and most urban district in Israel. To the best of our knowledge, this is the first study using a large, population-based cohort to study elderly non-consulters. We found that approximately 5% of this population (excluding the home-care population) can be classified as non-consulters with regard to PCP visits. Not consulting a PCP, however, did not necessarily mean having no contact with the Israeli public health system: 66% of the non-consulters used at least one healthcare service. Non-consulters have a higher probability for being women, older, not married, and from a lower socio-economic neighborhood. However, we do not think that the lower socio-economic status presents a financial barrier to PCP visits, since every Israeli resident is covered by a national health insurance from birth to death, and a PCP visit requires only a symbolic fee (~$1.5 per 3 months). Moreover, some indigent populations are exempt even from this copayment.

Our findings suggest that most elderly non-consulters enjoy better health than consulters: they have substantially lower hospitalization rates and seem to suffer less from chronic diseases. Of course, the lower documented rates of chronic diseases for non-consulters might, in part, be an artifact caused by their lack of PCP visits. However, other health indicators inspected in this study, such as hospitalizations and use of other hospital services – which may be considered indicators for major or urgent medical problems – are less prone to suffer from this artifact. In addition, even patients who do not regularly visit PCPs are included in morbidity registers. The retrospective character of the registers and the automated computerized update reduce the probability of undocumented chronic diseases.

Our findings are in line with previous studies from Israel [14] and elsewhere [5-7,15] that also tend to conclude that the health status of non-consulters is better compared to the general elderly population. In addition, undiagnosed medical problems might be common in the very elderly population, regardless of whether or not they visited their PCP [16]. Although further research is needed to more firmly establish the need, or lack thereof, for a proactive approach in elderly non-consulters, we believe that, assuming good mental health and no serious immobility, the decision not to visit a doctor is part of the patient's autonomy regarding his or her health.

## Conclusions

As a group, Israeli elderly who do not visit PCP seem to enjoy better health, and the need to reach out and screen them for various conditions is questionable, given the high workload on the Israeli PCP.

## Competing interests

The authors declare that they have no competing interests.

## Authors’ contributions

Dr. Eshel initiated the study, drafted the paper and made the final writing. Dr. Raz planned and conducted data analysis. Dr. Chodick revised the draft paper and suggested interpretation. Dr. Guindy initiated the study. All authors helped in the final writing. All authors read and approved the final manuscript.

## Authors’ information

Nira Eshel, MD, is a family physician in Maccabi Healthcare Services, Jerusalem, Israel. Raanan Raz, PhD, is currently a research fellow at Harvard School of Public Health Department of Environmental Health. In the past he served as a researcher in the Medical Informatics Department, Maccabi Healthcare Services, and in the Child Development Center at Sheba Medical Center. Gabriel Chodick, PhD, is Unit Head, Epidemiology and Database Research, Maccabi Healthcare Services. He is a senior lecturer at the School of Public Health in Tel Aviv University. Dr. Chodick has an adjunct position at the Division of Cancer Epidemiology & Genetics at the NIH. Michal Guindy, MD, is medical director of Central District, Maccabi Healthcare Services, and serves as the head of the patient safety program in Tel Aviv University, Israel.
