# Correction: Characteristics of the elderly who do not visit primary care physicians

**DOI:** 10.1186/2045-4015-2-46

**Published:** 2013-12-11

**Authors:** Nira Eshel, Raanan Raz, Gabriel Chodick, Varda Shalev, Michal Guindy

**Affiliations:** 1 Maccabi Healthcare Services, Department of Family Medicine, Hadassah Haktana Clinic, 6 Chile St, Jerusalem 9683204, Israel; 2 Maccabi Healthcare Services, Medical Informatics Department, Tel-Aviv, Israel; 3 Maccabi Healthcare Services, Central District, Tel-Aviv, Israel

## Correction

While compiling the article describing Characteristics of the elderly who do not visit primary care physicians [1], one of the authors was inadvertently omitted from the author list. This author, Varda Shalev, has been included in the corrected author list above.

## Authors’ contributions

NE initiated the study, drafted the paper and made the final writing. RR planned and conducted data analysis. GC revised the draft paper and suggested interpretation. MG and VS initiated the study. All authors helped in the final writing. All authors read and approved the final manuscript.

## Authors’ information

Nira Eshel, MD, is a family physician in Maccabi Healthcare Services, Jerusalem, Israel. Raanan Raz, PhD, is currently a research fellow at Harvard School of Public Health Department of Environmental Health. In the past he served as a researcher in the Medical Informatics Department, Maccabi Healthcare Services, and in the Child Development Center at Sheba Medical Center. Gabriel Chodick, PhD, is Unit Head, Epidemiology and Database Research, Maccabi Healthcare Services. He is a senior lecturer at the School of Public Health in Tel Aviv University. Dr. Chodick has an adjunct position at the Division of Cancer Epidemiology & Genetics at the NIH. Varda Shalev MD is the director of Primary Care Division of Maccabi Health Care Services and teaches Medical Informatics at Tel Aviv University,Israel, Michal Guindy, MD, is medical director of Central District, Maccabi Healthcare Services, and serves as the head of the patient safety program in Tel Aviv University, Israel.
